# Phenotypic and molecular characterization of *Streptococcus pneumoniae* serotype 3 isolates from blood and respiratory samples in Canada: CANWARD 2007–21

**DOI:** 10.1093/jac/dkae272

**Published:** 2024-08-02

**Authors:** John J Schellenberg, Heather J Adam, Melanie R Baxter, James A Karlowsky, Alyssa R Golden, Irene Martin, George G Zhanel

**Affiliations:** Department of Medical Microbiology and Infectious Diseases, Max Rady College of Medicine, Room 543-745 Bannatyne Avenue, Winnipeg, Manitoba, Canada R3E 0J9; Department of Medical Microbiology and Infectious Diseases, Max Rady College of Medicine, Room 543-745 Bannatyne Avenue, Winnipeg, Manitoba, Canada R3E 0J9; Clinical Microbiology, Shared Health, MS673-820 Sherbrook Street, Winnipeg, Manitoba, Canada R3A 1R9; Department of Medical Microbiology and Infectious Diseases, Max Rady College of Medicine, Room 543-745 Bannatyne Avenue, Winnipeg, Manitoba, Canada R3E 0J9; Department of Medical Microbiology and Infectious Diseases, Max Rady College of Medicine, Room 543-745 Bannatyne Avenue, Winnipeg, Manitoba, Canada R3E 0J9; Clinical Microbiology, Shared Health, MS673-820 Sherbrook Street, Winnipeg, Manitoba, Canada R3A 1R9; National Microbiology Laboratory, Public Health Agency of Canada, 1015 Arlington Street, Winnipeg, Manitoba, Canada R3E 3R2; National Microbiology Laboratory, Public Health Agency of Canada, 1015 Arlington Street, Winnipeg, Manitoba, Canada R3E 3R2; Department of Medical Microbiology and Infectious Diseases, Max Rady College of Medicine, Room 543-745 Bannatyne Avenue, Winnipeg, Manitoba, Canada R3E 0J9

## Abstract

**Background:**

Lower respiratory infections and invasive disease caused by *Streptococcus pneumoniae* serotype 3 remain major clinical challenges around the world, despite widespread availability of updated vaccines.

**Methods:**

As part of CANWARD, antimicrobial susceptibility testing and serotyping were performed on all *S. pneumoniae* isolates from 2007 to 2021. A subset of 226/264 (85.6%) serotype 3 isolates were selected for WGS to determine sequence type (ST)/clonal cluster (CC) and correspondence of antimicrobial resistance determinants (*erm*, *mefAE*, *tetM*, *cat*, *folA*, *folP*) with resistance phenotype.

**Results:**

Of the 3,039 *S. pneumoniae* isolates obtained from 2007 to 2021, 8.7% (*n* = 264) were serotype 3, with 64.0% of respiratory origin and 36.0% from blood. Of 226 sequenced serotype 3 isolates, 184 (81.4%) were ST180 (GPSC12). The proportion of ST8561 (single locus variant of ST180) increased from 7.2% to 16.6% during the study period. An increasing proportion of serotype 3 isolates had phenotypic resistance (*P* = 0.0007) and genetic resistance determinants (*P* = 0.004), comparing 2017–21 to 2007–11, largely due to a recently expanded ST180 clade with *cat*, *tetM* and *mef* determinants.

**Conclusions:**

*S. pneumoniae* serotype 3 from GPSC12 continues to dominate throughout Canada, with an increase in the proportion of ST8561. The proportion of serotype 3 isolates that are phenotypically resistant and with genetic resistance determinants is increasing over time, reflecting a global increase in GPSC12 genotypes with known resistance determinants. Phylogenomic characterization of isolates collected over time and from around the world may facilitate improved treatment and enhanced prevention strategies, including new vaccines with activity against *S. pneumoniae* serotype 3.

## Introduction

Severe infections caused by *Streptococcus pneumoniae*, including pneumonia and invasive pneumococcal disease (IPD), are among the earliest described of all infectious diseases,^1^ disproportionately affecting the youngest and oldest age groups, and those with specific risk factors. *S. pneumoniae* infections represent a majority of all bacterial lower respiratory infections around the world.^2,3^ The pathogenicity of *S. pneumoniae* is strongly related to its polysaccharide capsule, encoded by *cps* genes, with highly variant molecular composition and three-dimensional structure. Some serotypes are more strongly related to pneumonia and others to invasive disease, with serotype 3 recognized as one of the most virulent serotypes in terms of outcome severity.^4–6^

Early protection against *S. pneumoniae* serotype 3 was anticipated after its inclusion in the 23-valent pneumococcal polysaccharide vaccine (PPSV-23), widely available since the 1980s, and in the pneumococcal conjugate vaccines covering 13 or more serotypes (PCV13, PCV15 and PCV20), widely available since 2009. Although severe pneumococcal disease caused by most PCV13 serotypes has declined significantly for all age groups, serotype 3 continues to represent a significant proportion of invasive isolates.^5^ A recent study from Quebec, Canada showed little protection against serotype 3 IPD in any age group since PCV13 became available in that jurisdiction.^7^ The majority of studies in a recent meta-analysis confirmed the lack of PCV13 vaccine effectiveness against serotype 3.^8^

Globally, serotype 3 isolates mostly belong to sequence types (STs) in clonal complex 180 (CC180), which is part of Global Pneumococcus Sequence Cluster 12 (GPSC12).^9^ This phylogenetic group includes emerging subclades with distinct antimicrobial resistance (AMR) phenotypes.^5,10,11^ Members of our group and others have reported increased resistance in subclades of *S. pneumoniae* serotype 3 with specific genetic AMR determinants.^12,13^ Therefore, the goal of this study was phenotypic and molecular characterization of *S. pneumoniae* serotype 3 isolates from blood and respiratory samples, compared to other PCV13 serotypes (1, 4, 5, 6A, 6B, 7F, 9V, 14, 18C, 19A, 19F and 23F) and non-vaccine serotypes collected across Canada in the CANWARD study from 2007 to 2021.

## Materials and methods

### Bacterial isolates

As part of the CANWARD study, the Canadian Antimicrobial Resistance Alliance (CARA) partners with the Public Health Agency of Canada’s National Microbiology Laboratory (PHAC-NML) to work with tertiary-care medical centres in major population centres in 8 of the 10 Canadian provinces, as previously described.^14,15^ In this study, we compared *S. pneumoniae* isolates obtained from blood as well as respiratory samples obtained from sputum and bronchoalveolar lavages (BAL).

### Serotyping

Serotyping was performed by PHAC-NML on all *S. pneumoniae* isolates using the Quellung reaction with pool/group/type/factor commercial antisera (SSI Diagnostica; Statens Serum Institut, Copenhagen, Denmark), as previously described.^16^

### Antimicrobial susceptibility testing

All antimicrobial susceptibility testing was performed in-house, using custom broth microdilution panels prepared based on CLSI standard methods, including quality control, as previously described.^16^ Antimicrobial MIC interpretive standards were defined according to CLSI breakpoints^17^ for ceftriaxone (CEF), chloramphenicol (CHL), clarithromycin (CLR), clindamycin (CLD), doxycycline (DOX), levofloxacin (LEV), linezolid (LZD), penicillin (PEN), trimethoprim-sulfamethoxazole (SXT) and vancomycin (VAN). CHL susceptibility data were not collected prior to 2011. PEN resistance was defined as an MIC ≥2 mg/L.^16^

### Whole-genome sequencing and analysis

Isolates were sequenced by the PHAC-NML DNA Core facility using Illumina technology. Raw.fastq files (R1/R2) were processed and assembled using well-established in-house pipelines, including quality control using FastQC, contig assembly using Shovill, and annotation using prokka.^18^ Output.gff files were analysed using roary,^19^ with output core genome alignment (.aln) analysed using SNP-sites.^20^ Output alignments in.fasta format were used to create maximum likelihood phylogeny in Newick (.nhx) format using FASTTREE.^21^ The presence/absence of accessory genes in Newick format, also an output of roary, was used to estimate phylogenetic distance between isolates based on gene acquisition and deletion. Resulting trees were visualized with FigTree (http://tree.bio.ed.ac.uk/software/figtree/). Contigs were analysed using WADE (Whole-Genome Analysis for the Determination of Molecular Determinants; github.com/phac-nml/wade). The results of this custom pipeline include molecular serotyping, MLST, identification of AMR determinants and prediction of MIC, as previously described.^22,23^ ST results were compared to *S. pneumoniae* isolates deposited in the PubMLST database.^24^ Global Pneumococcus Sequence Cluster (GPSC) for each sequence type was accessed at https://www.pneumogen.net/gps (accessed 10 April 2024). All genome data generated as part of this study are available as part of Bioproject # PRJNA###### (The submission is still in progress).

### Statistical tests

Differences between clinical categories (Fisher’s exact test) were calculated using free online tools (www.medcalc.org).

## Results

### Profile of serotype 3 isolates from CANWARD

**Figure 1.**
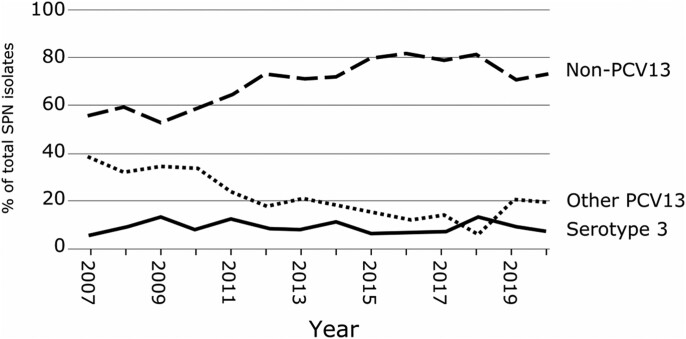
Proportion of total CANWARD *S. pneumoniae* isolates that were serotype 3 (solid line), other PCV13 serotypes 1, 4, 5, 6A, 6B, 7F, 9V, 14, 18C, 19A, 19F and 23F (short-dashed line) and non-PCV13 serotypes (long-dashed line).

**Figure 2.**
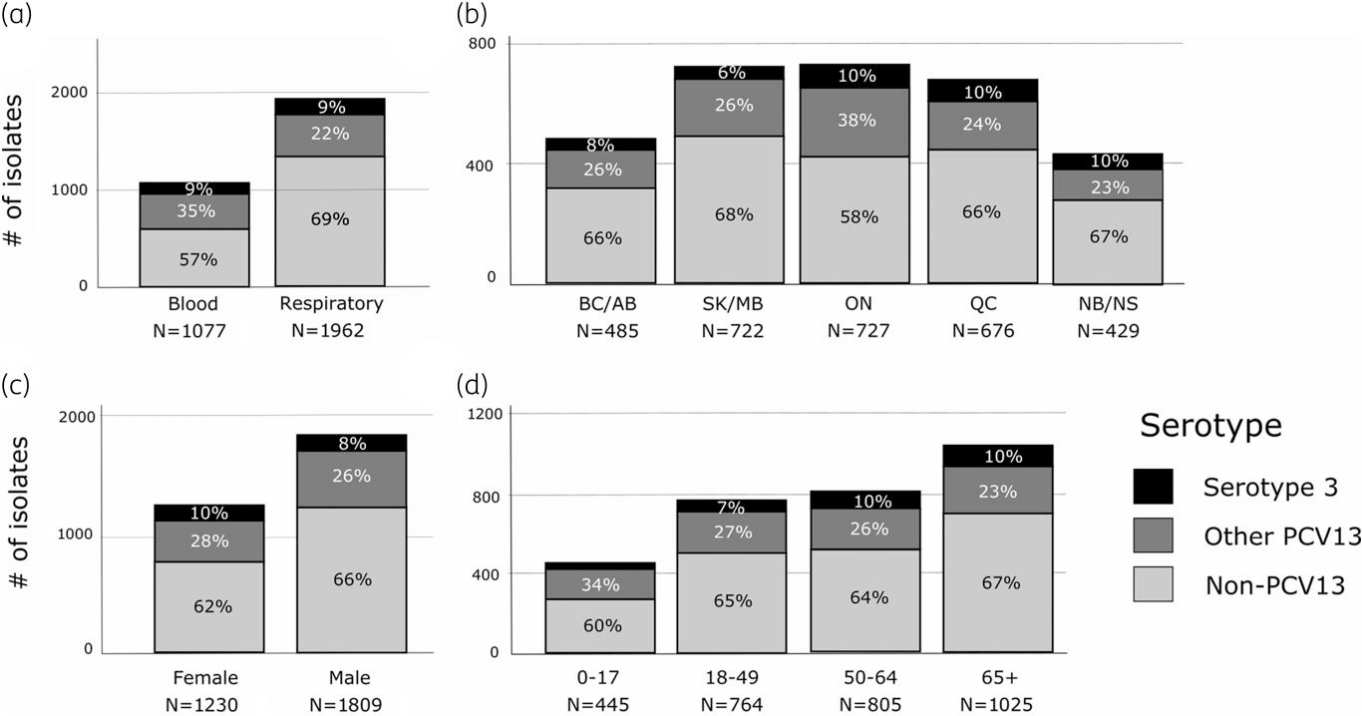
All *S. pneumoniae* isolates from CANWARD (*N* = 3,039), by serotype (serotype 3, other serotypes in PCV13 vaccine formulation and non-PCV13 serotypes), (a) isolate source, (b) province (BC/AB, British Columbia and Alberta; SK/MB, Saskatchewan and Manitoba; ON, Ontario; QC, Quebec; NB/NS, New Brunswick and Nova Scotia), (c) sex and (d) age. PCV, pneumococcal conjugate vaccine.

**Figure 3.**
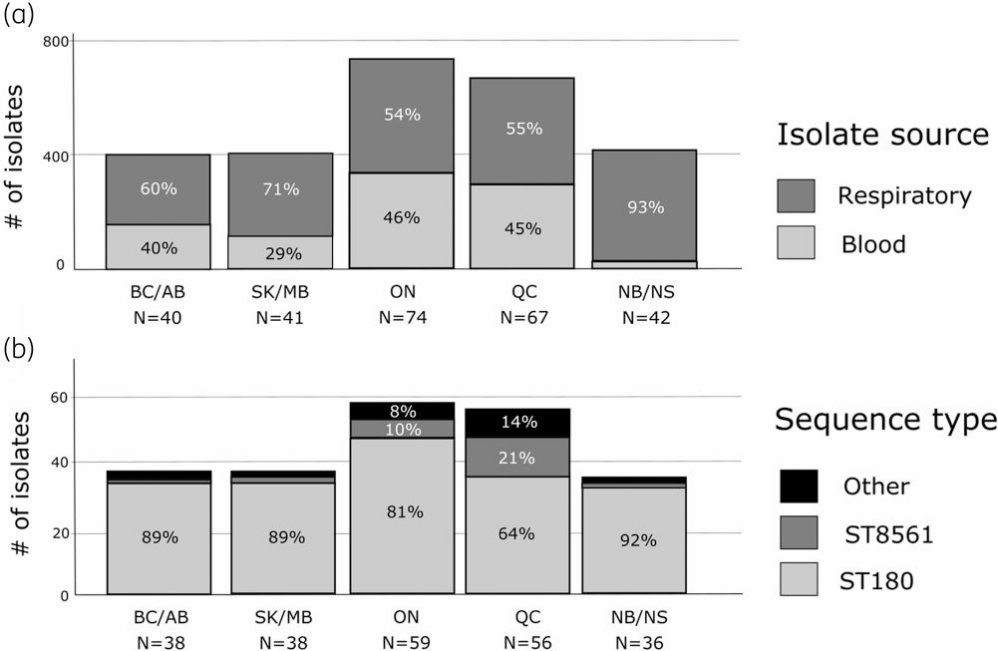
Regional distribution of *S. pneumoniae* serotype 3 isolates, by (a) isolate source (all serotype 3, *N* = 264), and (b) MLST from subset of serotype 3 with WGS (*N* = 226). (BC/AB, British Columbia and Alberta; SK/MB, Saskatchewan and Manitoba; ON, Ontario; QC, Quebec; NB/NS, New Brunswick and Nova Scotia).

A total of 56,952 clinical isolates were obtained from patients attending hospitals for care across Canada from 2007 to 2021. *S. pneumoniae* represented 5.3% of all bacterial isolates (Supplementary Table S1, available as Supplementary data at *JAC* Online). A total of 264 serotype 3 isolates made up 8.7% of all *S. pneumoniae*, ranging from 6% to 14% per year. The prevalence of other PCV13 serotypes (1, 4, 5, 6A, 6B, 7F, 9V, 14, 18C, 19A, 19F and 23F) declined while non-PCV13 serotypes increased over the study period (Figure 1, Supplementary Table S1). Most of the serotype 3 isolates were from respiratory samples (64.0%), with the remaining isolates from blood (36.0%). The majority of isolates were from males (53.8%), with 9.1% from those <18 years old, 21.6% from those aged 18 to 49 years and 69.3% from those aged 50 years and older (Supplementary Table S2). Isolates were received from all regions of Canada with the greatest numbers from Ontario (28.0%) and Quebec (25.4%), the most populous Canadian provinces (Supplementary Table S2). Respiratory isolates were significantly over-represented in serotype 3 compared to other PCV13 serotypes (64% versus 54%, *P* = 0.005 by Fisher’s exact test) (Supplementary Table S2). Limited regional variation was observed in terms of the proportion of serotype 3 from respiratory or blood sources or relative to other serotypes (Figure 2a and Figure 3a). Although the majority of *S. pneumoniae* isolates were from older age groups, the proportions of serotype 3, other PCV13 serotypes and non-PCV13 serotypes were similar regardless of sex or age (Figure 2c and d). Serotype 3 represented significantly more isolates from those aged 50 years and older, compared to younger age groups (10% versus 6.7%, *P* = 0.0003 by Fisher’s exact test) (Figure 2d, Supplementary Table S2).

### Profile of serotype 3 isolates over time

**Figure 4.**
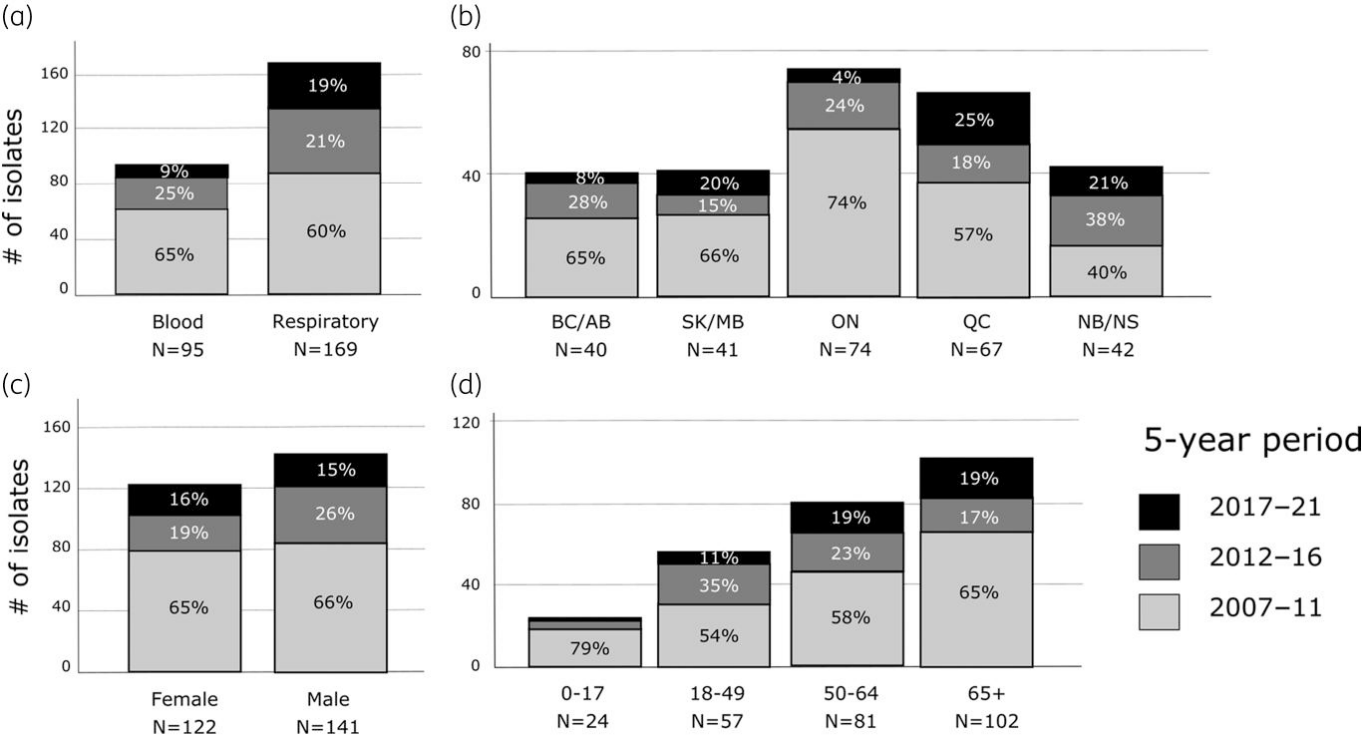
Cumulative *S. pneumoniae* serotype 3 isolates from CANWARD (*N* = 264) over time, by 5-year period (2007–11, 2012–16 and 2017–21), (a) by isolate source, (b) by province (BC/AB, British Columbia and Alberta; SK/MB, Saskatchewan and Manitoba; ON, Ontario; QC, Quebec; NB/NS, New Brunswick and Nova Scotia), (c) by sex, and (d) by age. PCV: pneumococcal conjugate vaccine.

When comparing cumulative isolates by 5-year time period, corresponding to pre-introduction/introduction of PCV-13 (2007–11), early-post-introduction (2012–16) and late-post-introduction (2017–21), a higher proportion of respiratory isolates than blood isolates were serotype 3 (19% versus 9%, *P* = 0.05 by Fisher’s exact test) in 2017–21 (Figure 4a, Supplementary Table S3). The 2017–21 prevalence of serotype 3 was higher in Quebec (25%), Nova Scotia/New Brunswick (21%) and Saskatchewan/Manitoba (20%), compared to British Columbia/Alberta (8%) and Ontario (4%) (Figure 4b, Supplementary Table S3). Similar proportions of isolates by time period were observed for males and females (Figure 4c), with a higher proportion in 2017–21 for older compared to younger age groups (19% versus 9%, *P* = 0.04 by Fisher’s exact test) (Figure 4d, Supplementary Table S3).

### AMR phenotype and genetic resistance determinants

Of 264 *S. pneumoniae* serotype 3 isolates identified in CANWARD, 230 had phenotypic AMR profiles (Table 1) and 226 underwent WGS and molecular profiling of AMR determinants (Supplementary Table S1). Of 136 isolates from 2007–11, only 21 isolates (all from 2011) had phenotypic CHL profiles available, therefore some CHL-resistant isolates from this time period were likely missed. A significantly higher proportion of isolates demonstrating any phenotypic resistance was observed in 2017–21 versus 2007–11 and 2012–16 combined (19.1% versus 3.2%, *P* = 0.0007 by Fisher’s exact test) (Table 1), including a higher proportion of isolates that were CHL-resistant (*P* = 0.008) and DOX-resistant (*P* = 0.001) compared to non-resistant isolates (Table 1). Of strains with phenotypic resistance to more than one antibiotic class (e.g. CHL/DOX, CHL/CLR/DOX, CHL/CLD/CLR/DOX), seven of eight (88%) isolates were from 2017 to 2021 (Table 1).

**Table 1.** AMR phenotype of 230 *S. pneumoniae* serotype 3 isolates by 5-year periods (2007–21)

	5-year periods		
	2007–11 (*N* = 136)	2012–16 (*N* = 53)	2017–21 (*N* = 41)
No resistance	132 (97.1%)	51 (96.3%)	33 (80.5%)
Any resistance^a^	4 (2.9%)	2 (3.7%)	8 (19.5%)
CHL-R^a^	—^b^	1 (1.9%)	6 (14.6%)
CLR-R	1 (0.7%)	—	4 (9.8%)
CLD-R	—	—	2 (4.9%)
DOX-R^a^	2 (1.4%)	2 (3.7%)	7 (17.1%)
Resistance Patterns			
CHL DOX	—	1 (1.9%)	4 (9.8%)
CLR DOX	—	—	1 (2.4%)
CHL CLR CLD DOX	—	—	2 (4.8%)
Intermediate Phenotypes			
CLR-I	3 (2.1%)	—	—
DOX-I	2 (1.4%)	2 (3.7%)	—
LEV-I	1 (0.7%)	—	—
PEN-I	1 (0.7%)	1 (1.9%)	1 (2.4%)
SXT-I	—	—	4 (9.8%)

CHL, chloramphenicol; CLR, clarithromycin; CLD, clindamycin; DOX, doxycycline; LEV, levofloxacin; PEN, penicillin; SXT, trimethoprim-sulfamethoxazole; I, Intermediate phenotype.

^a^Increased proportion of isolates with any resistant phenotype, CHL-R or DOX-R compared to no resistance is significant when comparing 2017–21 to 2007–11 and 2012–16 combined (*P* < 0.01 by Fisher’s exact test).

^b^For CHL resistance, *N* = 21, since CHL testing was only available for 21 isolates from 2007 to 2011.

Similarly, a significantly higher proportion of isolates with WGS were found to have any genotypic resistance determinant in 2017–21 versus 2007–11 and 2012–16 combined, (25% vs. 6.5%, *P* = 0.004 by Fisher’s exact test) (Table 2). Similar significant differences were observed for isolates with *erm* (*P* = 0.001), *tetM* (*P* = 0.002) and *cat* (*P* = 0.001), compared to isolates without these resistance determinants (Table 2). Overall concordance between phenotypic resistance and the presence of genetic resistance determinants is shown in Supplementary Table S5.

**Table 2.** MLST and molecular resistance determinants of 226 *S. pneumoniae* serotype 3 isolates with whole-genome sequences, 2007–21, by 5-year period

	5-year periods		
	2007–11 (*N* = 138)	2012–16 (*N* = 52)	2017–21 (*N* = 36)
**Resistance Determinants**			
Any determinant ^a^	9 (6.5%)	—	9 (25.0%)
*erm*+ ^a^	4 (3%)	—	6 (17%)
*mef*AE+	3 (2%)	—	2 (6%)
*tet*M+ ^a^	7 (5%)	—	7 (19%)
*cat*+ ^a^	4 (3%)	—	6 (17%)
*fol*A+	—	—	1 (3%)
*fol*P+	—	—	2 (6%)
*pbp*2X variant	2 (1%)	—	—
*gyr*A variant	1 (1%)	—	—
*par*C variant	1 (1%)	—	—
**MLST**			
ST180/CC180/GPSC12	118 (85.5%)	40 (77.4%)	26 (72.2%)
ST8561/CC180	10 (7.2%)	6 (11.3%)	6 (16.6%)
Others	10 (7%)	6 (11.3%)	4 (11.1%)

^a^Increased proportion of isolates with any resistance determinant, *erm*, *tetM* or *cat*, compared to no resistance, is significant when comparing 2017–21 to 2007–11 and 2012–16 combined (*P* < 0.01 by Fisher’s exact test).

### GPSC and resistance phenotype

All but one ST100 (GPSC3) isolate with phenotypic resistance and/or molecular resistance determinants belonged to ST180 (GPSC12). A higher proportion of non-resistant isolates from ST other than ST180 was observed in each successive 5-year period (from 14.5% to 22.6% to 27.8%, *P* > 0.05, non-significant by Fisher’s exact test) (Table 2). Other ST included the closely related ST8561 (single locus variant of ST180, *n* = 22), ST232 (GPSC83, *n* = 4, both blood and respiratory isolates), ST1116 (GPSC234, *n* = 3, blood isolates only). Single representatives belonging to 13 other STs were also observed, from GPSC83 (7 isolates), GPSC3 (2 isolates), GPSC135, GPSC44, GPSC51 and GPSC253 (1 isolate each). Of these, 10 were from respiratory samples and three from blood (Supplementary Table S4). ST8561 was particularly common in Quebec and Ontario (Figure 3b) and from respiratory sources (Supplementary Table S4).

### WGS-based phylogeny and serotype 3 subclades

**Figure 5.**
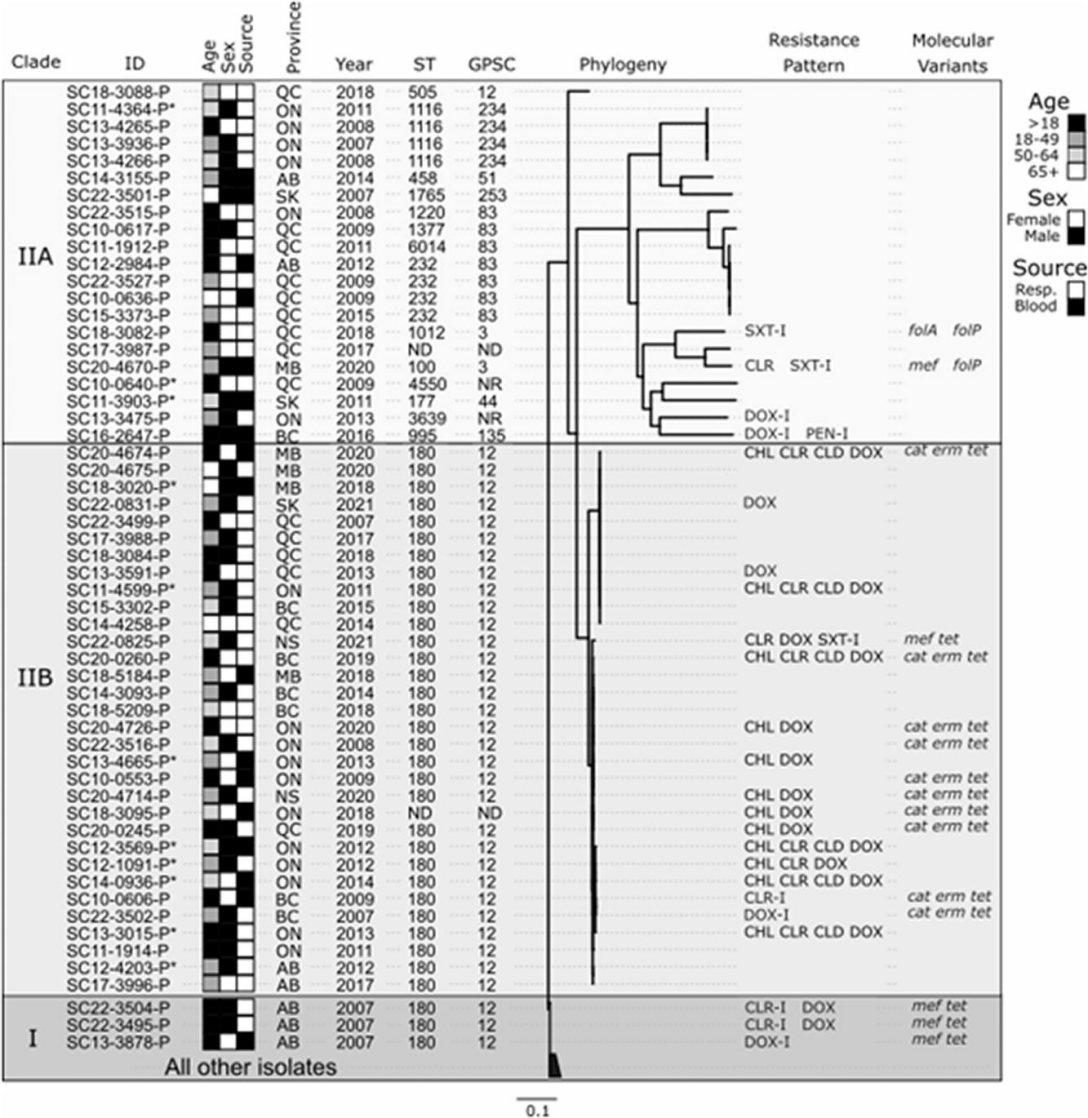
Core genome alignment-based phylogeny of 226 *S. pneumoniae* serotype 3 isolates sequenced in this study and 40 previously sequenced isolates (most closely related clade I isolates not shown, see Supplementary Figure S1 for accessory gene presence/absence-based phylogeny of all 267 isolates). *Branches with asterisks indicate previously published WGS by study co-authors. ST, Sequence Type determined by WGS; ND, ST Not Determined. GPSC, Global Pneumococcus Sequence Cluster; NR, No matching GPSC record for this ST. Resp., Respiratory isolates. BC, British Columbia; AB, Alberta; SK, Saskatchewan; MB, Manitoba; ON, Ontario; QC, Quebec; NB, New Brunswick; NS, Nova Scotia. CHL, chloramphenicol; CLR, clarithromycin; CLD, clindamycin; DOX, doxycycline; PEN, penicillin; SXT, trimethoprim-sulfamethoxazole; I, Intermediate phenotype.

Overall quality and coverage of assemblies resulting from sequencing runs was very high (Supplementary Figure S1). Phylogenetic analysis of the 226 genomes sequenced in this study showed two clades (I and II) with two subclades (IIA and IIB) (Figure 5, Supplementary Figure S2). Subclade I contained very little phylogenetic diversity (ST180 or single locus variants) but included a cluster of three *mef/tet* molecular variants that were non-susceptible to doxycycline (DOX-R or DOX-I) and two of which were also clarithromycin-intermediate (CLR-I). Subclade IIA contained multiple STs and extensive phylogenetic diversity, including a ST100 isolate that was CLR-R with a *mef* determinant and trimethoprim-sulfamethoxazole-intermediate (SXT-I). This isolate clustered with another SXT-I isolate from ST1012/GPSC3. Two more DOX-I isolates in Clade IIA were from ST995 (GPSC135, also penicillin-intermediate) and ST3639 (no GPSC). Subclade IIB contained most of the phenotypic resistance and molecular AMR determinants observed in this study, all of which belonged to a closely related cluster within ST180.

### Regional variation of isolate source and Mlst

Of 226 *S. pneumoniae* serotype 3 isolates with MLST, 66% were from respiratory samples and 34% were from blood, with similar proportions of ST180, ST8561 or other STs in each group (Supplementary Table S4). When comparing this group of *S. pneumoniae* isolates with public information regarding *S. pneumoniae* isolates in the PubMLST database,^24^ serotype 3 isolates were mostly from blood or other sterile sites (65%), compared to 35% from respiratory sites (Supplementary Table S4). Several STs identified in this study did not have other serotype 3 representatives in PubMLST, including ST100, ST995 and ST3639. A single respiratory serotype 3 isolate from Québec in 2017 had a novel MLST (no current representatives in PubMLST as of 10 April 2024).

The proportion of *S. pneumoniae* serotype 3 isolates from blood was highest in Ontario (46%) and Quebec (45%) and lowest in New Brunswick/Nova Scotia (7%) (Figure 4a). Isolates from ST8561 and other STs made up a higher proportion of isolates from Ontario (18%) and Quebec (35%) (Figure 3b). Most of the isolates with ST8561 or other STs from Quebec were collected in 2017–21, while those from other areas of the country were mostly collected in 2011–16, indicating a recent expansion of STs other than ST180 in Quebec.

## Discussion

Since serotype 3 capsular antigens are present in the current conjugated vaccines PCV13, PCV15 and PCV20, which are used worldwide in both children and adults,^25^ the ongoing prevalence of serotype 3 isolates has caused significant concern. Despite coverage by widely available vaccines, *S. pneumoniae* serotype 3 continues to be an important bacterial pathogen associated with invasive (e.g. bacteraemia, meningitis) and non-invasive (e.g. community-acquired respiratory tract) infections worldwide.^25^ Many pathogen and host factors predispose to severe *S. pneumoniae* infections and progression to invasive disease, with specific serotypes such as 3 and 19A associated with severe outcomes.^26–28^ The proportion of *S. pneumoniae* infections caused by serotype 3 has remained steady overall and increased in older age groups, in this study and in many other studies from Canada and around the world,^29–34^ indicating reduced or short-lived protection and/or lack of protection against colonization by vaccination.

Specific serotypes have been associated with colonization, mucosal diseases such as otitis media and pneumonia, severe outcomes including IPD and septic shock, as well as AMR and MDR.^4,13,25,27,35^ Recent research indicates that profuse production and release of serotype 3 capsule overwhelms the protective capacity of antibody from vaccines and allows the organism to escape neutrophil extracellular traps.^4^

In this study, we showed that serotype 3 continues to be very commonly associated with bacteraemia and respiratory tract infections (isolates obtained from sputum and BAL specimens in patients with respiratory tract infections) across Canada (Figures 1 and 2, Table S1), similar to many other studies around the world. Serotype 3 caused infections in all regions of the country, both sexes, all age groups and its prevalence did not change from 2007 to 2021 despite universal conjugate pneumococcal vaccination in children and widespread usage in adults at risk (Tables S2 and S3).^25,36^ Our findings are consistent with previous findings, which reported that serotype 3 was the most common invasive serotype reported across Canada from 2011 to 2020 as part of the CARA/PHAC-NML partnered surveillance study SAVE.^37,38^

The impact of vaccine formulations on serotype distribution through expansion of non-vaccine serotypes and/or capsular switching has been highly variable around the world.^30,39^ Direct and indirect protection by PCV13 against infection by serotype 3 has been limited,^8,40,41^ with rising rates of infections with serotype 3 observed globally. In the SAVE study, serotype 3 infections occurred in all age groups but increased in adults, particularly in the oldest age groups (those 50 years and older), indicating a lack of direct or indirect protection of older adults against this PCV13 serotype during the time period covered.^37^

We confirm previous observations of two subclades of ST180 (GPSC12), including a subclade with *ermB*/*tetM*/*cat* resistance determinants, also described in the SAVE study.^11,12,22^ These subclades make up the majority of isolates with increased phenotypic resistance and MDR observed in this study over the past five years, and have been well-described in isolate collections from around the world.^5,10,13^ A large WGS-based study including isolates from several countries highlighted the global distribution of ST180 subclades, corresponding to those observed in the current study.^10^ The isolates that we observed with *ermB/tetM/cat* determinants correspond with a subset of Clade II isolates from the USA and Hong Kong, increasingly prevalent in Asia and North America.^10^ The four isolates with *tetM/mef* determinants that we observed may correspond with *tetM *+ Clade 1b isolates in the international study.^10^ In a UK study of isolates from IPD patients, Clade II appeared in 2008, made up 5%–10% of all serotype 3 isolates until 2013–14, then rapidly increased to 30%–50% of serotype 3 isolates in 2017–18.^5^ In Colombia, subtyping analysis of 365 serotype 3 isolates collected between 1994 and 2015 by PFGE identified two subgroups with phenotypic resistance,^13^ likely corresponding to the subclades described in other studies.^10,11^ In Argentina, more than half of a subset of ST180 isolates (7/11) were tetracycline-resistant, indicating the presence of *tet* determinants consistent with previously described subclades.^42^

Increased phenotypic resistance in subclades of *S. pneumoniae* serotype 3 ST180 isolates in recent years reflects a wider trend of rapid pneumococcal evolution in response to clinical interventions, including antimicrobial use and vaccination.^43^ The majority of serotype 3 isolates sequenced in this study were ST180 (185/226 or 82.3%), including all but one isolate with any resistant phenotype. The remaining isolate with phenotypic resistance was a single ST100 (GPSC3) isolate from 2020 with a *mef* determinant and phenotypic resistance to clarithromycin, as well as a *folP* determinant and intermediate phenotype for SXT. Most of the isolates with ST100 in PubMLST are from serotype 33F, with no serotype 3 isolates yet included. Most isolates with ST1012 (GPSC3) in PubMLST are also from serotype 33F with only a single serotype 3 submitted with this ST to date. In this study, we observed a single ST1012 isolate with intermediate phenotypic resistance to SXT and *folA*/*folP* resistance determinants and a single ST995 (GPSC135) isolate with no resistance determinants and intermediate phenotype for DOX and PEN. All ST995 isolates so far in PubMLST are from serotype 16F. ST1377 (GPSC83, one isolate) has previously been described as a common ST for serotype 3 isolates in Canada, with one study finding that up to 5% of serotype 3 isolates in Ontario were ST1377.^44^ We also observed a single isolate with a unique ST that is a single locus variant of ST662 (GPSC3, with only serotype 3 isolates in PubMLST) and ST7192 (with only serotype 33F isolates in PubMLST).

*S. pneumoniae* is one of the earliest whole genomes sequenced.^45^ There is already a relatively long history of WGS pipelines for determining molecular serotype, isolate relatedness, AMR determinants and virulence factors for this organism,^44,46^ including the custom pipeline used in this study that was created by PHAC-NML.^47^ The results of the current analysis further previous observations of WGS-based concordance with phenotypic resistance, at least for some antibiotic classes (Supplementary Table 5). As WGS becomes less expensive and more rapid, instant reference to rich genotypic datasets to assist clinical decision-making is increasingly feasible.

Major strengths of the current study include the availability of isolates from both blood and respiratory samples (sputum and BAL) and robust nationwide distribution, providing insight into geographic variation of serotype 3 across Canada. A limitation is that there are a relatively smaller number of isolates available for WGS at later timepoints, including no isolates from 2012 to 2016 that were found to have resistance determinants. Since WGS was not conducted on all serotype 3 isolates with phenotypic resistance from this time period, and almost all isolates with phenotypic resistance were also positive for one or more genetic resistance determinants (Table 2), it is likely that sequencing more isolates would result in better representation over time.

In summary, 8.7% of all *S. pneumoniae* collected in the CANWARD study were serotype 3 and the prevalence did not change over time. WGS of serotype 3 isolates demonstrated that although ST180 (GPSC12) continues to dominate throughout Canada (in both blood and respiratory isolates), the single locus variant ST8561 is increasing over time. Subclades of ST180 were associated with a significantly higher proportion of serotype 3 being phenotypically resistant and possessing genetic resistance determinants over the timeframe of this study. Our findings support the well-established need for a vaccine that can provide universal protection against all serotypes by targeting non-capsular targets.^48^

## Supplementary Material

dkae272_Supplementary_Data

## Member laboratories of Canadian Antimicrobial Resistance Alliance

Vancouver Hospital, Vancouver, BC; University of Alberta Hospital, Edmonton, AB; Royal University Hospital, Saskatoon, SK; Health Sciences Centre, Winnipeg, MB; London Health Sciences Centre, London, ON; Mount Sinai Hospital/University Health Network, Toronto, ON; The Ottawa Hospital, Ottawa, ON; St Michael’s Hospital, Toronto, ON; St Joseph’s Hospital, Hamilton, ON; Children’s Hospital of Eastern Ontario, Ottawa, ON; Hôpital Maisonneuve Rosemont, Montreal, QC; Montreal General Hospital, Montreal, QC; Royal Victoria Hospital, Montreal, QC; CHRTR Pavillon Ste. Marie, Trois Rivières, QC; Centre Hospitalier Universitaire de Sherbrooke, Sherbrooke, QC; Cité de la Santé, Laval, QC; L’Hôtel Dieu de Québec, Québec, QC; South East Health Care Corp., Moncton, NB; and Queen Elizabeth II Health Sciences Centre, Halifax, NS.

## Supplementary data

Figures S1 and S2 and Tables S1–S5 are available as Supplementary data at *JAC* Online.
