# Supplementary material for: Phenotypic and molecular characterization of Streptococcus pneumoniae serotype 3 isolates from blood and respiratory samples in Canada: CANWARD 2007–21
Source: J Antimicrob Chemother. 2024 Aug 2;79(10):2653–61. doi: 10.1093/jac/dkae272 (PMC11442004; doi:10.1093/jac/dkae272)
Supplement: dkae272_Supplementary_Data [file dkae272_supplementary_data.docx]

**Supplementary Materials**

*Supplementary Table S1:* Numbers of all pathogens, *S. pneumoniae* isolates, *S. pneumoniae* serotype 3 isolates, and *S. pneumoniae* serotype 3 isolates that received WGS in CANWARD, 2007-2021.

| **Year** | **All Pathogens in CANWARD** | **All SPN in CANWARD** | **SPN Serotype 3** | **% of all SPN Serotype 3 Isolates Sequenced** |
| --- | --- | --- | --- | --- |
| **2007** | 7,718 | 695 (9%) | 46 (7%) | 41 (89%) |
| **2008** | 5,283 | 531 (10%) | 48 (9%) | 39 (81%) |
| **2009** | 5,373 | 212 (4%) | 29 (14%) | 23 (79%) |
| **2010** | 4,960 | 224 (5%) | 18 (8%) | 17 (94%) |
| **2011** | 3,785 | 190 (5%) | 22 (12%) | 18 (82%) |
| **2012** | 2,802 | 140 (5%) | 12 (8%) | 8 (67%) |
| **2013** | 3,511 | 188 (5%) | 15 (8%) | 12 (87%) |
| **2014** | 3,172 | 157 (5%) | 17 (11%) | 16 (94%) |
| **2015** | 3,206 | 136 (4%) | 8 (6%) | 8 (100%) |
| **2016** | 3,126 | 124 (4%) | 8 (6%) | 8 (100%) |
| **2017** | 3,420 | 117 (3%) | 8 (7%) | 6 (75%) |
| **2018** | 2,963 | 124 (4%) | 16 (13%) | 13 (81%) |
| **2019** | 3,069 | 81 (3%) | 7 (9%) | 7 (100%) |
| **2020** | 2,231 | 81 (3%) | 6 (7%) | 6 (100%) |
| **2021** | 2,333 | 39 (2%) | 4 (10%) | 4 (100%) |
| **Total:** | **56,952** | **3,039 (5.3%)** | **264 (8.7%)** | **226 (85.6%)** |

SPN, *S. pneumoniae*

*Supplementary Table S2:* Isolate source and patient demographics of all *S. pneumoniae* collected by CANWARD from 2007 to 2021, PCV13 serotypes other than serotype 3, serotype 3 isolates, and serotype 3 isolates that received WGS.

|  | **Study Group** | | | |
| --- | --- | --- | --- | --- |
|  | **All SPN**  **(n=3,039)** | **PCV13 serotypes other than serotype 3 (n=811)** | **Serotype 3 (n=264)** | **Serotype 3 sequenced (n=226)** |
| **Source *** |  |  |  |  |
| Blood | 1,077 (35.5%) | 373 (46.0%) | 95 (36.0%) | 77 (34.1%) |
| Respiratory | 1,962 (64.5%) | 438 (54.0%) | 169 (64.0%) | 149 (65.9%) |
| **Sex** |  |  |  |  |
| Female | 1,230 (40.5%) | 340 (41.9%) | 122 (46.2%) | 105 (46.5%) |
| Male | 1,809 (59.5%) | 471 (58.1%) | 142 (53.8%) | 121 (53.5%) |
| **Age (years)** |  |  |  |  |
| 0 to 1 | 134 (4.4%) | 43 (5.3%) | 5 (1.9%) | 3 (1.3%) |
| 2 to 5 | 165 (5.4%) | 57 (7.0%) | 9 (3.4%) | 9 (4.0%) |
| 6 to 17 | 146 (4.8%) | 53 (6.5%) | 10 (3.8%) | 8 (3.5%) |
| 18 to 49 | 764 (25.1%) | 210 (25.9%) | 57 (21.6%) | 44 (19.5%) |
| 50 to 64 | 805 (26.5%) | 212 (26.1%) | 81 (30.7%) | 72 (31.9%) |
| 65 or more | 1,025 (33.7%) | 236 (29.1%) | 102 (38.6%) | 89 (39.4%) |
| **Province** |  |  |  |  |
| British Columbia | 186 (6.1%) | 52 (6.4%) | 17 (6.4%) | 16 (7.0%) |
| Alberta | 299 (9.8%) | 74 (9.1%) | 23 (8.7%) | 22 (9.7%) |
| Saskatchewan | 423 (13.9%) | 110 (13.6%) | 21 (8.0%) | 19 (8.4%) |
| Manitoba | 299 (9.8%) | 81 (10.0%) | 20 (7.6%) | 19 (8.4%) |
| Ontario | 727 (23.9%) | 232 (28.6%) | 74 (28.0%) | 59 (26.0%) |
| Quebec | 676 (22.2%) | 165 (20.3%) | 67 (25.4%) | 56 (24.7%) |
| New Brunswick | 202 (6.6%) | 37 (4.6%) | 19 (7.2%) | 16 (7.1%) |
| Nova Scotia | 227 (7.5%) | 60 (7.4%) | 23 (8.7%) | 19 (8.4%) |

* Proportion of respiratory isolates in serotype 3 is higher than the proportion from isolates in other PCV13 serotypes: 1, 4, 5, 6A, 6B, 7F, 9V, 14, 18C, 19A, 19F, and 23F (p=0.005 by Fisher’s exact test).

*Supplementary Table S3:* Isolate source and patient demographics of all *S. pneumoniae* serotype 3 isolates in CANWARD, 2007-2021 (n=264)

|  | **5-year periods** | | |
| --- | --- | --- | --- |
|  | **2007-2011**  **(n=163)** | **2012- 2016**  **(n=60)** | **2017-2021 (n=41)** |
| **Source *** |  |  |  |
| Blood | 62 (38.0%) | 24 (40.0%) | 9 (22.0%) |
| Respiratory | 101 (62.0%) | 36 (60.0%) | 32 (78.0%) |
| **Sex** |  |  |  |
| Female | 79 (48.5%) | 23 (38.3%) | 20 (48.7%) |
| Male | 84 (51.5%) | 37 (61.7%) | 21 (51.3%) |
| **Age (years) *** |  |  |  |
| 0 to 1 | 4 (2.5%) | 1 (1.7%) | 0 |
| 2 to 5 | 6 (3.7%) | 3 (5.0%) | 0 |
| 6 to 17 | 9 (5.5%) | 0 | 1 (2.4%) |
| 18 to 49 | 31 (19.0%) | 20 (33.3%) | 6 (14.6%) |
| 50 to 64 | 47 (28.8%) | 19 (31.7%) | 15 (36.7%) |
| 65 or more | 66 (40.5%) | 17 (28.3%) | 19 (46.3%) |
| **Province** |  |  |  |
| British Columbia | 8 (4.9%) | 7 (11.7%) | 2 (4.9%) |
| Alberta | 18 (11.0%) | 4 (6.7%) | 1 (2.4%) |
| Saskatchewan | 14 (8.6%) | 4 (6.7%) | 3 (7.3%) |
| Manitoba | 13 (8.0%) | 2 (3.3%) | 5 (12.2%) |
| Ontario | 55 (33.7%) | 15 (25.0%) | 4 (9.8%) |
| Quebec | 38 (23.3%) | 12 (20.0%) | 17 (41.5%) |
| New Brunswick | 4 (2.5%) | 10 (17.6%) | 5 (12.2%) |
| Nova Scotia | 13 (8.0%) | 6 (10.0%) | 4 (9.8%) |

* Proportion of respiratory isolates versus blood isolates and those aged 50 years or older versus 49 years and younger is significantly increased in 2017-2021 compared to 2007-2011 and 2012-2016 combined (p = 0.05 and p = 0.04 respectively by Fisher’s exact test).

*Supplementary Table S4:* CANWARD *S. pneumoniae* serotype 3 isolates by source and MLST, compared to PubMLST isolates

|  | **SPN serotype 3** | | | **PubMLST SPN - serotype 3** | | | **PubMLST SPN - Other** | | | **PubMLST alleles** | | | | | | |
| --- | --- | --- | --- | --- | --- | --- | --- | --- | --- | --- | --- | --- | --- | --- | --- | --- |
| **MLST** | **Total** | **Resp.** | **Blood** | **Total** | **Resp.** | **Blood** | **Total** | **Resp.** | **Blood** | ***aroE*** | ***gdh*** | ***gki*** | ***recP*** | ***spi*** | ***xpt*** | ***ddl*** |
| ST180 | 184 | 119 (65%) | 65 (35%) | 1,119 | 389 (35%) | 730 (65%) | 4 | 2 (50%) | 2 (50%) | 7 | 15 | 2 | 10 | 6 | 1 | 2 |
| ST8561 | 22 | 16 (73%) | 6 (27%) | 1 | - | 1 | - | - | - | 7 | 15 | 372 | 10 | 6 | 1 | 22 |
| ST11873 | 1 | 1 | - | 1 | - | 1 | - | - | - | 7 | 15 | 2 | 10 | 6 | 676 | 2 |
| ST232 | 4 | 1 (25%) | 3 (75%) | 16 | - | 16 | - | - | - | 13 | 9 | 15 | 14 | 10 | 16 | 1 |
| ST1116 | 3 | 3 | - | 2 | - | 2 | - | - | - | 1 | 26 | 28 | 11 | 13 | 1 | 14 |
| ST100 | 1 | - | 1 | - | - | - | 108 | 45 (42%) | 63 (58%) | 5 | 12 | 29 | 12 | 9 | 39 | 18 |
| ST458 | 1 | 1 | - | 192 | 60 (31%) | 132 (69%) | 2 | - | 2 | 2 | 32 | 9 | 47 | 6 | 21 | 17 |
| ST505 | 1 | 1 | - | 86 | 51 (59%) | 35 (41%) | 1 | 1 | - | 46 | 8 | 2 | 10 | 6 | 1 | 22 |
| ST995 | 1 | 1 | - | - | - | - | 5 | 1 (20%) | 4 (80%) | 1 | 5 | 4 | 4 | 6 | 58 | 8 |
| ST1012 | 1 | - | 1 | 1 | - | 1 | 32 | 15 (47%) | 17 (53%) | 2 | 5 | 29 | 18 | 42 | 3 | 18 |
| ST1220 | 1 | 1 | - | 16 | 4 (25%) | 12 (75%) | - | - | - | 26 | 1 | 15 | 14 | 9 | 16 | 19 |
| ST1377 | 1 | - | 1 | 25 | 5 (20%) | 20 (80%) | - | - | - | 13 | 84 | 15 | 14 | 10 | 16 | 19 |
| ST1765 | 1 | 1 | - | 5 | 2 (40%) | 3 (60%) | - | - | - | 2 | 32 | 54 | 47 | 114 | 115 | 17 |
| ST3639 | 1 | 1 | - | - | - | - | - | - | - | 1 | 2 | 29 | 18 | 6 | 20 | 18 |
| ST6014 | 1 | 1 | - | 3 | 2 (66%) | 1 (33%) | - | - | - | 194 | 9 | 15 | 14 | 10 | 16 | 1 |
| ST10038 | 1 | 1 | - | 1 | - | 1 | - | - | - | 309 | 15 | 2 | 10 | 6 | 1 | 22 |
| Unique* | 1 | - | - | - | - | - | - | - | - | 5 | 35 | 29 | 12 | 9 | 16 | 18 |
| Total | 226 | 149 (66%) | 77 (34%) | 1,469 | 513 (35%) | 956 (65%) | 163 | 64 (39%) | 99 (61%) |  | | | | | | |

* No exact match, close to ST662 (with *xpt* allele 39), and ST7192 (with *xpt* allele 45); SPN, *S. pneumoniae;* Resp., respiratory

*Supplementary Table S5: Concordance between WGS and phenotypic AMR*

|  |  |  | Isolates positive for genetic determinants (% of row total) | | | | | |
| --- | --- | --- | --- | --- | --- | --- | --- | --- |
| Phenotype | | Tested Isolates* | *ermB* | *mefAE* | *tetM* | *folA* | *folP* | *cat* |
| CHL | S | 95 | - | 2 (2%) | 1 (1%) | 1 (1%) | 2 (2%) | - |
|  | R | 6 | 6 (100%) | - | 6 (100%) | - | - | 6 (100%) |
| CLR | S | 188 | 7 (4%) | 1 (>1%) | 8 (4%) | 1 (>1%) | 1 (>1%) | 7 (4%) |
|  | I | 3 | 1 (33%) | 2 (67%) | 3 (100%) | - | - | 1 |
|  | R | 5 | 2 (40%) | 2 (40%) | 3 (60%) | - | 1 (20%) | 2 (40%) |
| CLD | S | 194 | 8 (4%) | 5 (3%) | 12 (6%) | 1 (>1%) | 2 (1%) | 8 (4%) |
|  | R | 2 | 2 (100%) | - | 2 (100%) | - | - | 2 (100%) |
| DOX | S | 182 | 3 (2%) | 1 (>1%) | 3 (2%) | 1 (>1%) | 2 (1%) | 3 (2%) |
|  | I | 4 | 1 (25%) | 1 (25%) | 2 (50%) | - | - | 1 (25%) |
|  | R | 10 | 6 (60%) | 3 (30%) | 9 (90%) | - | - | 6 (60%) |
| PEN | S | 192 | 10 (5%) | 5 (3%) | 14 (7%) | 1 (>1%) | 2 (1%) | 10 (5%) |
|  | I | 3 | - | - | - | - | - | - |
| SXT | S | 195 | 10 (5%) | 3 (2%) | 13 (6%) | - | - | 10 (5%) |
|  | I | 3 | - | 2 (67%) | 1 (33%) | 1 (33%) | 2 (67%) | - |

S, Susceptible. I, Intermediate. R, Resistant. * Note that total tested isolates are different for each antimicrobial agent due to missing data.

*Supplementary Figure S1: Technical data from WGS runs (N=226), Mb: Megabases*


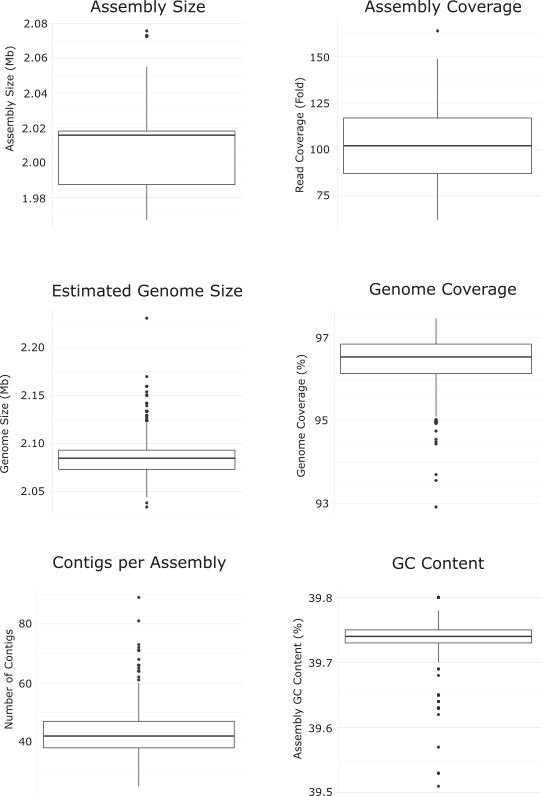


*Supplementary Figure S2:* Accessory gene presence/absence-based phylogeny of 226 isolates from this study, with 40 previously published WGS. Three subclusters contain most of the ST diversity and all of the phenotypic resistance/molecular AMR determinants. Shaded branches indicate previously published WGS by study co-authors, which are extended and confirmed by these results. Resp. = Respiratory isolates. BC: British Columbia, AB: Alberta, SK: Saskatchewan, MB: Manitoba, ON: Ontario, QC: Quebec, NB: New Brunswick, NS: Nova Scotia. CHL: chloramphenicol, CLR: clarithromycin, CLD, clindamycin, DOX: doxycycline, LEV: levofloxacin, PEN: penicillin, SXT: trimethoprim-sulfamethoxazole, I: Intermediate phenotype.
